# Влияние костно-анаболической терапии на параметры костного ремоделирования и плотность кости у гериатрических пациентов с остеопорозом и синдромом падений

**DOI:** 10.14341/probl13079

**Published:** 2022-04-04

**Authors:** Н. О. Ховасова, Е. Н. Дудинская, А. В. Наумов, О. Н. Ткачева, Л. В. Мачехина, Ю. С. Онучина

**Affiliations:** Кафедра болезней старения, Российский национальный исследовательский медицинский университет им. Пирогова; Лаборатория заболеваний костно-мышечной системы, Российский геронтологический научно-клинический центр; Кафедра болезней старения, Российский национальный исследовательский медицинский университет им. Пирогова; Лаборатория возрастных метаболических и эндокринных нарушений, Российский геронтологический научноклинический центр; Кафедра болезней старения, Российский национальный исследовательский медицинский университет им. Пирогова; Лаборатория заболеваний костно-мышечной системы, Российский геронтологический научно-клинический центр; Кафедра болезней старения, Российский национальный исследовательский медицинский университет им. Пирогова; Кафедра болезней старения, Российский национальный исследовательский медицинский университет им. Пирогова; Лаборатория возрастных метаболических и эндокринных нарушений, Российский геронтологический научноклинический центр; Кафедра болезней старения, Российский национальный исследовательский медицинский университет им. Пирогова; Лаборатория возрастных метаболических и эндокринных нарушений, Российский геронтологический научноклинический центр

**Keywords:** остеопороз, падения, переломы, костно-анаболическая терапия, терипаратид

## Abstract

**ОБОСНОВАНИЕ:**

ОБОСНОВАНИЕ. Пожилые люди с тяжелым остеопорозом — наиболее уязвимая группа гериатрических пациентов. Им показано назначение антиостеопоротической терапии, которая должна быть эффективной и безопасной. Терипаратид продемонстрировал снижение риска переломов, прирост минеральной плотности кости (МПК). В России применение терипаратида в гериатрической популяции исследовано крайне скудно.

**ЦЕЛЬ:**

ЦЕЛЬ. Оценить клиническое течение, параметры костного метаболизма и эффективность костно-анаболической терапии у пациентов пожилого и старческого возраста с тяжелым остеопорозом и синдромом падений.

**МАТЕРИАЛЫ И МЕТОДЫ:**

МАТЕРИАЛЫ И МЕТОДЫ. В продольное проспективное исследование включены 100 пациентов 60 лет и старше с тяжелым остеопорозом, перенесших одно или более падений в течение последнего года. Всем пациентам назначались препараты кальция и витамина D и костно-анаболическая терапия (терипаратид 20 мкг ежедневно подкожно). Длительность наблюдения составила 24 мес и включала 3 визита: скрининг, через 12 и 24 мес. Эффективность костно-анаболической терапии проводили на основе оценки частоты новых переломов, редукции болевого синдрома, изменений МПК по данным рентгеновской денситометрии, динамике маркеров костного метаболизма.

**РЕЗУЛЬТАТЫ:**

РЕЗУЛЬТАТЫ. Все пациенты имели тяжелый остеопороз и отягощенный коморбидный статус, перенесли падение в течение последнего года, а также низкоэнергетические переломы в прошлом. Каждый третий пациент имел вертебральный перелом, каждый пятый — перелом проксимального отдела бедренной кости. До начала исследования антиостеопоротическую терапию получал 61 пациент. В течение наблюдения умерли 4 пациентки, исследование завершили 96 пациентов. На фоне терапии терипаратидом получено уменьшение количества новых случаев низкоэнергетических переломов и числа пациентов с хронической болью. Прирост МПК отмечен в поясничном отделе позвоночника через 24 мес и в шейке бедра — через 12 мес. Отрицательной динамики МПК зафиксировано не было. Также через 12 мес отмечено увеличение P1NP и С-концевого телопептида коллагена 1 типа, через 24 мес — остеокальцина и С-концевого телопептида.

**ЗАКЛЮЧЕНИЕ:**

ЗАКЛЮЧЕНИЕ. Применение терипаратида может быть рекомендовано как эффективная интервенция по лечению тяжелого остеопороза у гериатрических пациентов с синдромом падений.

## Abstract (en)

**BACKGROUND:**

BACKGROUND: Older adults with severe osteoporosis are the most vulnerable group of geriatric patients. They are shown the purpose of anti-osteoporotic therapy, which should be effective and safe. Teriparatide showed a decrease in the risk of fractures, an increase in BMD. In Russia, the use of teriparatide in the geriatric population is extremely scarce.

**AIM:**

AIM: assess clinical course, bone metabolism parameters and efficacy of bone-anabolic therapy in elderly and senile patients with severe osteoporosis and falls.

**MATERIALS AND METHODS:**

MATERIALS AND METHODS: The longitudinal prospective study included 100 patients 60 years and older with severe osteoporosis who had one or more falls within the last year. All patients were prescribed calcium and vitamin D preparations and bone-anabolic therapy (teriparatide 20 mg daily subcutaneously). The duration of follow-up was 24 months and included 3 visits: screening, at 12 and 24 months. The effectiveness of bone-anabolic therapy was carried out on the basis of assessing the frequency of new fractures, reduction of pain, changes in BMD according to X-ray densitometry, dynamics of bone metabolism markers.

**RESULTS:**

RESULTS: All patients had severe osteoporosis and aggravated comorbidity status, suffered a fall within the last year, and also low-energy fractures in the past. One in three patients had a vertebral fracture, one in five had a proximal femoral fracture. Prior to the start of the study, 61 patients received antiosteoporotic therapy. During the follow-up, 4 patients died, 96 patients completed the study. Against the background of teriparatide therapy, a decrease in the number of new cases of low-energy fractures and the number of patients with chronic pain was obtained. An increase in BMD was noted in the lumbar spine after 24 months and in the femoral neck after 12 months. There was no negative dynamics of the BMD. Also after 12 months, an increase in P1NP and C-terminal telopeptide of collagen type 1 was noted, after 24 months — osteocalcin and C-terminal telopeptide.

**CONCLUSION:**

CONCLUSION: The use of teriparatide can be recommended as an effective intervention to treat severe osteoporosis in geriatric patients with falls.

Тяжелый остеопороз — это остеопороз с уже имеющимся в анамнезе патологическим переломом: тела позвонка(ов), переломом бедренной кости или множественными переломами независимо от степени снижения минеральной плотности кости (МПК) по данным рентгеновской денситометрии [1]. Пожилые люди с тяжелым остеопорозом относятся к наиболее уязвимой группе гериатрических пациентов. Тяжелый остеопороз ассоциирован с рядом гериатрических синдромов, высоким риском падений и повторных переломов, хронической болью, потерей автономности, инвалидизацией и повышенным риском смерти [2–5].

Несмотря на угрожающие последствия, далеко не все пациенты с диагностированным остеопорозом и даже пациенты с уже случившимся низкоэнергетическим переломом получают антиостеопоротическую терапию [6]. Более того, данные Соединенных Штатов Америки демонстрируют снижение частоты лечения антиостеопоротическими препаратами после случившегося перелома бедренной кости с 41% в 2001 г. до 21% в 2010 г. [7]. Такая ситуация может быть объяснена низкой комплаентностью пациентов пожилого возраста, снижением когнитивных и социальных функций, отсутствием понимания в необходимости проведения длительной терапии. Кроме того, для лечения пациентов, перенесших низкоэнергетические переломы, требуются значительные финансовые средства. По данным Соединенных Штатов Америки ежегодные траты на лечение остеопоротических переломов совпадают или даже превышают ежегодные затраты на лечение инфаркта миокарда, инсульта, рака молочной железы [8]. В Европейском союзе в 2010 г. на лечение низкоэнергетических переломов было потрачено более 37 млрд евро. По прогнозам к 2025 г. эти затраты увеличатся еще на 25% [9].

К признанным фактора риска переломов относятся пожилой возраст, низкая МПК, падения [10]. Однако самым сильным фактором риска последующего перелома является предыдущий перелом [11]. В связи с чем лечение остеопороза необходимо инициировать сразу после перелома (если лечение до этого не проводилось) или продолжать даже если перелом уже случился.

В антиостеопоротической терапии выделяют 2 группы лекарственных препаратов: антирезорбтивные и костно-анаболические. Препараты первой группы снижают костную резорбцию, тогда как препараты второй группы — стимулирует образование новой костной ткани, изменяя плотность и архитектонику кости. К антирезорбтивным препаратам относятся бисфосфонаты и ингибиторы RANKL. К костно-анаболическим препаратам относятся терипаратид и абалопаратид. Последний в России не зарегистрирован. Эти препараты не считаются средствами первой линии для большинства пациентов из-за их высокой стоимости [12]. Однако, согласно Российским клиническим рекомендациям по остеопорозу 2021 г. терипаратид рекомендован для предупреждения патологических переломов и прибавки МПК у женщин в постменопаузе с компрессионными переломами тел позвонков, для лечения остеопороза у мужчин и в качестве первой линии терапии — у пациентов с остеопорозом с двумя и более переломами тел позвонков, при неэффективности предшествующей терапии [13].

Терипаратид продемонстрировал свою эффективность в виде снижения риска переломов, прироста МПК и ускоренного заживления переломов в ряде зарубежных исследований [14–18]. В России эффективность терипаратида оценивалась в единичных исследованиях. В связи с чем нами было запланировано и выполнено исследование по оценке эффективности терапии терипаратидом в гериатрической популяции, страдающих тяжелым остеопорозом и перенесших падения.

## ЦЕЛЬ ИССЛЕДОВАНИЯ

Оценить клиническое течение тяжелого остеопороза, параметры костного метаболизма и эффективность костно-анаболической терапии у пациентов пожилого и старческого возраста с остеопорозом и синдромом падений.

## МАТЕРИАЛЫ И МЕТОДЫ

Место и время проведения исследования

Пациенты наблюдались амбулаторно в Российском геронтологическом научно-клиническом центре ФГАОУ ВО «РНИМУ им. Н.И. Пирогова» Минздрава России в период октябрь 2018 — март 2021 гг.

Изучаемые популяции (одна или несколько)

В одноцентровое проспективное одновыборочное нерандомизированное интервенционное исследование были включены 110 пациентов 60 лет и старше с тяжелым остеопорозом, перенесшие одно или более падений в течение последнего года.

Критерии включения:

Критерии невключения:

Длительность наблюдения составила 24 мес. За это время осуществлялось 3 визита.

На 1-м визите (старт исследования) проводился скрининг пациента по критериям включения и исключения и при соответствии первым и отсутствии последних, подписывалось информационное согласие на участие в исследовании. У всех пациентов осуществлялся сбор анамнеза, в том числе лекарственного с уточнением о приеме антиостеопоротической терапии, клинический осмотр. Всем пациентам было проведено лабораторное и инструментальное обследование на базе лаборатории Российского геронтологического научно-клинического центра ФГАОУ ВО РНИМУ им. Н.И. Пирогова Минздрава России.

Перечень основных исследований включал следующие.

В случае развития тяжелых нежелательных реакций введение препарата прекращалось, и пациент исключался из исследования. В течение первых трех месяцев 3 пациента исключены из исследования по причине развития тяжелых нежелательных явлений: сильная плохо купируемая боль в области перелома (n=1), ортостатическая гипотония с падением (n=1), выраженная боль и судороги в мышцах (n=1). В итоге анализ проводился на 100 пациентах.

2-й визит выполнялся через 12 мес от исходного и включал оценку эффективности костно-анаболической терапии. Оценивались частота новых переломов и падений на фоне терапии, клинические симптомы (наличие и интенсивность болевого синдрома), изменения в динамике МПК по данным рентгеновской денситометрии, оценивалась динамика лабораторных показателей и маркеров костного метаболизма.

3-й визит (завершение исследования) проводился через 24 мес от исходного и набором опций повторял 2-й визит. На данном визите также отменялась костно-анаболическая терапия, и пациенту рекомендовалось продолжение лечения антирезорбтивными препаратами под наблюдением специалиста по остеопорозу.

Статистический анализ

База данных создана в программе IBM® SPSS® Statistics version 23.0 (SPSS Inc., США). Вид распределения количественных переменных анализировали при помощи одновыборочного критерия Колмогорова–Смирнова. Количественные данные представлены как M±SD, где M — среднее, SD — стандартное отклонение; при сильном отклонении от нормальности данные представлены одновременно как M±SD и Ме (25%; 75%), где Ме — медиана, 25% и 75% — 25-й и 75-й процентили. Качественные данные представлены в виде абсолютных чисел и относительных частот (%).

Для межгрупповых сравнений использовали критерии Манна–Уитни, Краскела–Уоллиса, χ2 Пирсона и двусторонний точный тест Фишера. Различия считали значимыми при двустороннем значении р<0,05.

Этическая экспертиза

Исследовательская работа была одобрена независимым этическим комитетом ФГАОУ ВО «РНИМУ им. Н.И. Пирогова» Минздрава России (Протокол заседания НЭК No 16 от 18.09.2018). Все участники исследования подписали информированное письменное согласие.

## РЕЗУЛЬТАТЫ

Общая характеристика пациентов, включенных в исследование, представлена в табл. 1.

Все пациенты имели отягощенный соматический статус. Структура и распространенность коморбидной патологии представлены в табл. 2.

Индекс коморбидности Чарльсон составил 6,43±1,64. По поводу коморбидной патологии пациенты принимали гипотензивную терапию, статины, антиагреганты, антикоагулянты. Гипогликемическая терапия была представлена метформином (n=11), вилдаглиптином (n=5), гликлазидом (n=3), инсулином (n=6). Менопаузальную гормональную терапию получала одна пациентка, препараты левотироксина — 5.

Все пациенты, включенные в исследование, имели тяжелый остеопороз и перенесли падение в течение последнего года. Среднее количество падений за год составило 1,46±0,87, 2 и более падений случилось у 42%. 10-летний риск переломов по алгоритму FRAX представлен в табл. 3, из которой видно, что две трети пациентов имели высокий 10-летний риск переломов.

Все пациенты ранее перенесли низкоэнергетические переломы, их характеристика представлена в табл. 4.

**Table:** Таблица 1. Общая характеристика пациентов (n=100)

Характеристика	Значение	%
Возраст, лет	75,3±8,0	
Мужчины, n	2	2
Женщины, n	98	98
Рост, см	156,6±8,3	
Вес, кг	63,2±12,8	
ИМТ, кг/м2	25,8±4,8	
ИМТ менее 20 кг/м2	6	6
ИМТ 20–30 кг/м2	70	70
ИМТ 30–35 кг/м2	19	19
ИМТ 35–40 кг/м2	5	5
Курение (n)	6	6
Ранняя менопауза (n)	5	5,1

**Table:** Таблица 2. Структура и распространенность коморбидной патологии (n=100)

Заболевание	n
Артериальная гипертония	41
ИБС	18
Цереброваскулярная болезнь	10
Сахарный диабет	21
Остеоартрит	14
Узловой зоб	12
Анемия	12
Хроническая обструктивная болезнь легких	7
Язвенная болезнь желудка	4
Мочекаменная болезнь	4
Болезнь Паркинсона	2
Подагра	1

**Table:** Таблица 3. Оценка 10-летнего риска остеопоретических переломов по FRAX (n=100)

Заболевание	n
FRAX общий	25,1±9,5
FRAX бедро	11,3±9,1
Низкий риск по FRAX (n)	2
Средний риск по FRAX (n)	26
Высокий риск по FRAX (n)	72

**Table:** Таблица 4. Характеристика остеопоротических переломов и их последствий (n=100)

Характеристика	n
Среднее количество переломов	2,6±1,7
Количество пациентов со множественными переломами	81
Переломы тел позвонков	58
Среднее количество переломов тел позвонков	2,2±1,2
Чрескожная вертебропластика	6
Перелом проксимального отдела бедра	21
Операции по поводу перелома проксимального отдела бедра	7
Другие переломы	57
Операции остеосинтеза	4
Болевой синдром после перелома	78
Интенсивность боли, ВАШ (баллы)	7 (7;8)
Затруднения передвижения	19
Усиление кифоза грудного отдела позвоночника	15
Снижение роста на 4 см	36

Большая часть (81%) пациентов имели множественные переломы, максимальное количество переломов у одного пациента — 13. Каждый третий пациент имел вертебральный перелом, в большинстве случаев — не одного, а нескольких позвонков. У ряда пациентов имели клинические маркеры вертебральных переломов: снижение роста более чем на 4 см в течение жизни (n=36), усиление кифоза грудного отдела позвоночника (n=15). Вертебропластика проведена у 6 (10,3%) человек. Каждый пятый пациент имел перелом проксимального отдела бедренной кости, и лишь у трети (33,3%) из них проводилось оперативное лечение. В половине случаев диагностированы другие переломы, наиболее частые из них — перелом лучевой кости в типичном месте и перелом плечевой кости. 78% пациентов имели хронический болевой синдром с высокой интенсивностью по ВАШ, каждый пятый пациент испытывал затруднения при передвижении.

До начала исследования антиостеопоротическую терапию получал 61 пациент. Антирезорбтивную терапию бисфосфонатами получали 48 человек, деносумабом — 23 пациента, стронция ранелат — 2 человека. Фоновую терапию препаратами кальция получали 69 пациентов, препаратами витамина D3 — 72 пациента. На фоне вышеописанной терапии произошло 35 патологических перелома: 24 перелома (10 — вертебральные, 4 — проксимального отдела бедра, 10 — другие невертебральные) случились у пациентов, принимающих бисфосфонаты, 3 перелома (1 — вертебральный, 2 — невертебральные) — у пациентов, получающих деносумаб, 1 перелом (вертебральный) — на фоне приема стронция ранелата и 7 переломов (3 — вертебральные, 1 — проксимального отдела бедра, 3 — другие невертебральные) — у пациентов, получающих только препараты кальция и витамина D3.

В течение первого года умерла одна пациентка, в течение второго года — еще три пациентки, что при сравнении не имело статической значимости. Во всех случаях причиной смерти была декомпенсация коморбидной патологии у старых пациентов. Переломы не были непосредственной причиной смерти. Суммарно исследование завершили 96 пациентов.

За время терапии терипаратидом произошло 32 новых перелома — 28 переломов в первые 4 месяца костно-анаболической терапии, 4 перелома — на 13–18 месяцах терапии.

Всем пациентам была назначена костно-анаболическая терапия Терипаратидом, эффективность которой оценивалась через 12 и 24 месяца. Результаты представлены в табл. 5, 6, 7.

**Table:** Таблица 5. Клиническая эффективность костно-анаболической терапии терипаратидом (n=100)

Характеристика	Старт исследования (1), n=100	12 месяцев (2), n=99	24 месяца (3), n=97	р	р1–2	р1–3	р2–3
Количество новых переломов	32	6	3	<0,001	<0,001	<0,001	0,28
Количество человек, перенесших падения	100	33	28	<0,001	<0,001	<0,001	0,50
Болевой синдром (n)	78	19	16	<0,001	<0,001	<0,001	0,71
Интенсивность болевого синдрома, ВАШ (баллы)	7 (7;8)	4 (3;5)	3,5 (2;4)	0,005	0,022	<0,001	0,79
Затруднения при передвижении	19	12	11	0,266			
Смерть		1	3	0,363			

**Table:** Таблица 6. Динамика МПК на фоне костно-анаболической терапии терипаратидом (n=100)

Характеристика, M ± SD	Старт исследования (1), n=100	12 мес (2), n=99	24 мес (3), n=97	Δ 1–2	Δ 1–3	Δ 2–3
Т-критерий L1–L4	-2,97±1,27	-2,63±1,08	-1,72±1,7	0,34 [ -0,01; 0,77]	1,25 [ 0,45; 2,05]	0,91 [ 0,09; 1,73]
Т-критерий, худший по позвоночнику	-3,49±1,75	-3,09±1,0	-2,38±1,59	0,4 [ -0,13; 0,93]	1,11 [ 0,19; 2,03]	0,71 [-0,19; 1,61]
Т-критерий, шейка бедра	-2,67±0,82	-1,96±0,94	-2,32±0,78	0,71 [ 0,37; 1,05]	0,35 [ -0,03; 0,73]	-0,36 [ -0,8; 0,08]
Т-критерий, total hip	-2,49±1,07	-1,9±1,41	-2,04±1,18	0,59 [ 0,03; 1,15]	0,45 [ -0,17; 1,07]	-0,14 [ -0,91; 0,63]
				Δ% 1–2	Δ% 1–3	Δ% 2–3
BMD L1–L4	0,817±0,152	0,833±0,221	0,991±0,226	-1,8 [ -13,8; 10,6]	21,3 [ 4,6; 38,4]	23,5 [ 3,5; 45,5]
BMD, шейка бедра	0,653±0,118	0,726±0,147	0,915±0,869	11,3 [ 0,8; 22,1]	40,1 [ -29,9; 110,4]	25,9 [ -37,2; 89,8]
BMD, total hip	0,685±0,139	0,773±0,203	0,727±0,141	12,9 [ -1; 27,1]	6,1 [ -6,9; 19,4]	-6 [ -20,1; 9,2]

 

**Table:** Таблица 7. Динамика лабораторных маркеров костного ремоделирования на фоне костно-анаболической терапии (n=100)

Характеристика, M ± SD	Старт исследования (1), n=100	12 мес (2), n=99	24 мес (3), n=97	Δ% 1–2	Δ% 1–3	Δ% 2–3
N-терминальный пропептид проколлагена 1 типа (P1NP), нг/мл	64,16±49,03	121,23±88,43	88,79±50,05	88,9 [ 18,2; 175,2]	38,4 [ -13,2; 101,3]	-26,8 [ -54,8; 8,2]
Остеокальцин, нг/мл	20,70±17,61	24,51±15,65	44,46±26,16	18,4 [ -32,1; 87]	114,8 [ 19,2; 243,1]	97,5 [ 13,8; 204,6]
Щелочная фосфатаза, Ед/л	123,8±87,88	135,5±56,1	117,66±107,8	9,5 [ -18; 41,3]	-5 [ -51,1; 45]	-13,2 [ -55; 31,7]
С-концевой телопептид коллагена 1 типа, нг/мл	0,332±0,29	0,679±0,4	0,826±0,42	104,5 [ 15; 233,5]	148,8 [ 21,1; 324,5]	21,6 [ -34,2; 87,6]
Витамин D3, нг/дл	27,74±16,9	35,87±11,8	43,09±25,7	9,2 [ -0,14; -18,2]	13,7 [ -3,3; -18,2]	7,2 [ -17,9; 3,5]

Полученные данные демонстрируют достоверное уменьшение количества новых случаев низкоэнергетических переломов, частоты падений, а также статистически значимое уменьшение числа пациентов, испытывающих хронический болевой синдром, и интенсивность боли с сильной до умеренной.

Прирост МПК был отмечен в поясничном отделе позвоночника с достоверными изменениями через 24 месяца и в шейке бедра — через 12 мес применения терипаратида. Положительная динамика МПК была отмечена у 41 испытуемого через 12 мес наблюдения и у 20 человек через 24 мес (р<0,001 для тренда; при попарном сравнении на старте исследования и через 12 мес р<0,001, через 12 и 24 мес р=0,002). Отрицательной динамики МПК зафиксировано не было.

На фоне проводимой костно-анаболической терапии через 12 мес отмечено статистически значимое увеличение P1NP и С-концевого телопептида коллагена 1-го типа, через 24 мес — остеокальцина и С-концевого телопептида. Все пациенты в течение всего периода исследования получали препараты кальция и витамина D3, на фоне чего через 12 мес достоверно увеличилась концентрация витамина D3 и достигла в среднем нормальных значений. Через 24 мес лечения сохранялась тенденция к увеличению концентрации витамина D3.

Таким образом, показана клиническая, рентгенологическая и лабораторная эффективность препарата терипаратид в лечении тяжелого остеопороза у гериатрических пациентов, перенесших падения.

Тяжелые нежелательные явления (сильная плохо купируемая боль в области перелома, ортостатическая гипотония с падением, выраженная боль и судороги в мышцах) на фоне терапии, потребовавшие ее отмены, развились у 3 (2,9%) пациентов. Этим пациентам препарат терипаратид был отменен, и пациенты были исключены из исследования. У 11 (10,7%) пациентов отмечены нетяжелые нежелательные реакции, такие как тошнота (n=3), головокружение (n=2), ортостатическая гипотония (n=2), судороги в мышцах (n=1), одышка (n=1), тахикардия (n=1), транзиторная гиперкальциемия (n=1). Все эти нежелательные реакции развились в первые 3 месяца применения препарата, не были тяжелыми, что не потребовало отмены терапии.

## ОБСУЖДЕНИЕ

Репрезентативность выборок

Набор участников исследования проводился на базе федерального научного центра. В исследование вошли пациенты, обратившиеся амбулаторно в Российский геронтологический научно-клинический центр ФГАОУ ВО «РНИМУ им. Н.И. Пирогова» Минздрава России

Сопоставление с другими публикациями

Целью нашего исследования явилась оценка эффективности костно-анаболической терапии у пациентов пожилого и старческого возраста с остеопорозом и синдромом падений. Большинство пациентов, включенных в исследование, были женщины (98%) в возрасте 75,3±8,0 года, с высоким индексом коморбидности Чарльсон (6,43±1,64 балла). В нашем ранее опубликованном исследовании мы также получили, что у пациентов с остеопорозом в сравнении с пациентами без него индекс коморбидности достоверно выше [5].

Все пациенты имели в анамнезе низкоэнергетические переломы. Некоторые исследования свидетельствуют, что компрессионные переломы позвонков являются самым частым осложнением остеопороза [19]. Самыми частыми из всех перенесенных переломов у наших испытуемых были именно вертебральные переломы, в ряде случаев — нескольких позвонков. Этот феномен описан как «эффект домино»: перелом одного позвонка повышает вероятность для последующих переломов других позвонков в 5 раз и переломов других локализаций в 2–3 раза [20]. Каждый пятый испытуемый имел перелом проксимального отдела бедренной кости, и лишь трети (33,3%) из них проводилось оперативное лечение. Известно, что всем пациентам с патологическим переломом проксимального отдела бедренной кости рекомендуется госпитализация и хирургическое лечение в течение 48 часов [13, 21, 22]. Однако, в реальной клинической практике мы наблюдаем значительно более редкую частоту оперативных вмешательств, так же, как и назначение антиостеопоротической терапии. Несмотря на наличии тяжелого остеопороза антиостеопоротическую терапию до начала исследования получали далеко не все пациенты (61%).

Эффективность терипаратида была оценена в нашем исследовании на основании частоты новых переломов, прироста МПК и динамики маркеров костного ремоделирования. Полученные данные показали статистически значимое уменьшение количества новых случаев любых низкоэнергетических переломов через 12 мес лечения терипаратидом. Через 24 мес количество переломов также уменьшилось, но эти изменения не показали достоверной разницы. Снижение частоты переломов на фоне костно-анаболической терапии было показано в ряде исследований. Например в исследовании, в котором изучали данные Medicare и Medicaid Services США 1 278 296 женщин в возрасте 65 лет и старше, получавших различные антиостеопоротические препараты, в том числе терипаратид (n=20 610). Оказалось, что через 12 мес лечения было отмечено снижение переломов любой локализации, а также вертебральных и невертебральных переломов среди пациентов, получавших деносумаб, золедроновую кислоту, пероральные бисфосфонаты и терипаратид. При этом у пациентов, принимавших терипаратид, максимальное снижение риска было показано для вертебральных переломов (72%; ДИ 69%–75%) [23]. Метаанализ, включивший 107 исследований суммарно 193 987 женщин (возраст 66 лет; длительность наблюдения в среднем 28 мес), также показал значительное снижение переломов позвонков и внепозвоночных переломов при приеме терипаратида [24]. В сравнительном исследовании женщин с постменопаузой и тяжелым остеопорозом, получающих терипаратид и ризедронат, было показано большее снижение риска переломов позвонков и меньшее количество случившихся новых переломов среди пациенток в группе терипаратида [18]. Результаты еще одного мета-анализа показали, что применение терипаратида ассоциировано со снижением риска переломов позвонков (RR 0,57, 95% ДИ 0,35–0,93, р=0,024). Кроме того, на фоне терапии терипаратидом установлен прирост МПК в поясничном отделе позвоночника через 6, 12 и 18 мес в сравнении с бисфосфонатами (р<0,05) и МПК в шейке бедренной кости в течение 18 мес (р<0,05) [25]. В нашем исследовании статистически значимый прирост МПК был отмечен в несколько более поздние сроки: в поясничном отделе позвоночника через 24 месяца, а в шейке бедра — через 12 месяцев применения терипаратида.

Также наше исследование продемонстрировало, что через 12 мес терапии терипаратидом достоверно уменьшилось как число пациентов, испытывающих болевой синдром, так и интенсивность боли (с сильной до умеренной), через 24 мес костно-анаболической терапии такого эффекта отмечено не было. Ранее в метаанализе 4 исследований было показано статистически значимое снижение случаев боли в спине у пациентов, получавших терипаратид, по сравнению с плацебо и с антирезорбтивной терапией [26]. Peschalis E.P. и соавт. наблюдали у женщин в постменопаузе, получавших терипаратид, снижение сильных и умеренных болей в спине на 57% [27].

Американская ассоциация клинической химии и Национальный альянс здоровья костей США определяют основным маркером костной резорбции C-концевой телопепид коллагена 1 типа, а маркером образования кости — PINP [28]. Существуют доказательства, что повышенные уровни маркеров резорбции костей и пониженные уровни маркеров формирования костей связаны с повышенным риском переломов [29]. В ходе исследования мы оценивали маркеры костного ремоделирования. На фоне проводимой костно-анаболической терапии через 12 мес отмечено статистически значимое увеличение P1NP и С-концевого телопептида коллагена 1 типа, через 24 мес — остеокальцина и С-концевого телопептида. Известно, что PINP увеличивается через несколько месяцев после начала лечения терипаратидом, что с одной стороны используется для мониторинга эффективности лечения и с другой стороны является предиктором прироста МПК [30]. Повышение С-концевого телопептида коллагена 1-го типа может быть объяснено одновременным увеличением резорбции костей, которое наблюдается при лечении терипаратидом [31].

Согласно клиническим рекомендациям по остеопорозу 2021 г. все препараты для лечения остеопороза рекомендуется назначать в сочетании с препаратами кальция и колекальциферола [13]. Известно, что препараты кальция и витамина D3 оказывают положительное влияние на мышечную массу, силу и баланс, снижая риск падений и переломов [32, 33]. Все наши пациенты в течение всего периода исследования получали препараты кальция и витамина D3, на фоне чего зафиксировано достоверно увеличение концентрация витамина D3.

Клиническая значимость результатов

Полученные данные продемонстрировали эффективность и безопасность костно-анаболической терапии в лечении тяжелого остеопороза у гериатрических пациентов с синдромом падений. На фоне применения терипаратида показаны: уменьшение количества новых случаев любых низкоэнергетических переломов, статистически значимый прирост МПК в поясничном отделе позвоночника через 24 мес, в шейке бедра — через 12 мес проводимой терапии, положительная динамика маркеров костного ремоделирования и уменьшение числа пациентов, испытывающих боль и снижение ее интенсивности. Невысокая частота нежелательных побочных реакций, большая часть из которых нетяжелая, развивается и купируется в первые месяцы применения препарата, позволяет рекомендовать костно-анаболическую терапию не только как эффективную, но и безопасную, что играет важную роль в выборе лечебной стратегии у гериатрических пациентов.

Ограничения исследования

Большая временная часть исследования пришлась на период пандемии, вызванной SARS-CoV-2. Противоэпидемические ограничительные мероприятия касались в первую очередь пациентов пожилого и старческого возраста, что ограничивало их привычную повседневную физическую, когнитивную и социальную активность, что, возможно, могло повлиять на результаты и нашего исследования.

Направления дальнейших исследований

Считаем целесообразным проведение исследований по изучению влияния антиостеопоротической терапии на параметры гериатрического статуса (функциональный, когнитивный) с целью сохранения автономности пожилых людей, улучшения их качества и продолжительности жизни.

## ЗАКЛЮЧЕНИЕ

У гериатрических пациентов с тяжелым остеопорозом и синдромом падений применение костно-анаболической терапии показало снижение количества новых случаев любых низкоэнергетических переломов, прирост МПК в поясничном отделе позвоночника через 24 мес, в шейке бедра — через 12 мес проводимой терапии. Кроме того, снизилось количество пациентов, испытывающих хронический болевой синдром и интенсивность боли, а также отмечена положительная динамика маркеров костного ремоделирования. Все это позволяет считать применение терипаратида как эффективную интервенцию по лечению тяжелого остеопороза у гериатрических пациентов с синдромом падений.

## ДОПОЛНИТЕЛЬНАЯ ИНФОРМАЦИЯ

Источники финансирования. Исследование было выполнено в рамках Государственного задания «Оптимизация диагностики и ведения сочетанных заболеваний опорно-двигательного аппарата у пациентов пожилого и старческого возраста с целью сохранения автономности» № 69.7-2021.

Конфликт интересов. Авторы декларируют отсутствие явных и потенциальных конфликтов интересов, связанных с содержанием настоящей статьи.

Участие авторов. Ховасова Н.О. — существенный вклад в концепцию и дизайн исследования, в получение, анализ данных или интерпретацию результатов, написание статьи; Дудинская Е.Н. — существенный вклад в получение, анализ данных или интерпретацию результатов, внесение в рукопись существенной правки с целью повышения научной ценности статьи; Наумов А.В. — существенный вклад в концепцию и дизайн исследования, в получение, анализ данных или интерпретацию результатов, написание статьи; Ткачева О.Н. — существенный вклад в получение, анализ данных или интерпретацию результатов, внесение в рукопись существенной правки с целью повышения научной ценности статьи; Мачехина Л.В. — существенный вклад в получение, анализ данных или интерпретацию результатов; Онучина Ю.С. — существенный вклад в получение, анализ данных или интерпретацию результатов.

Все авторы одобрили финальную версию статьи перед публикацией, выразили согласие нести ответственность за все аспекты работы, подразумевающую надлежащее изучение и решение вопросов, связанных с точностью или добросовестностью любой части работы.
